# Impact of adjuvant therapy on survival in esophageal cancer patients after neoadjuvant therapy investigated by a population based cohort study

**DOI:** 10.1038/s41598-026-39930-5

**Published:** 2026-04-13

**Authors:** Jia-Lei Huang, Zi-Xin Lin, Ke-Yi Zeng, Zheng Lu, Xuan-Ping Lin, He-Rui Wang, Yong-Wei Xie, Jing-Rong Yang

**Affiliations:** 1 Department of Clinical Medicine, Fujian Medical University, Fuzhou, 350122 Fujian China; 2 Academy of Integrative Medicine, Fujian University of Traditional Chinese Medicine, Fuzhou, 350122 Fujian China; 3 The First Clinical College of Fujian Medical University, Fuzhou, 350005 China; 4 Department of Neurology, The First Affiliated Hospital, Fujian Medical University, Fuzhou, 350005 China; 5 Fuzong Clinical Medical College of Fujian Medical University & 900th Hospital of Joint Logistics Support Force, No.156 Northwest Second Ring Road, Fuzhou, 350025 Fujian China

**Keywords:** Esophageal cancer, Adjuvant therapy, Neoadjuvant therapy, Survival, SEER, Cancer, Oncology

## Abstract

While surgical intervention administered after neoadjuvant therapy represents the standard treatment in esophageal cancer, the survival benefit conferred by adjuvant therapy administered postoperatively among this patient cohort continues to be controversial. Using real-world data, 6141 esophageal cancer patients administered neoadjuvant treatment preoperatively between 2007 and 2021 were categorized into adjuvant therapy group (*n* = 1116) or no adjuvant therapy group(*n* = 5025). No significant differences in overall survival (OS) or cancer-specific survival (CSS) were observed between groups before or after propensity score matching (PSM), and adjuvant therapy was not identified as an independent prognostic factor. However, subgroup analyses revealed important variations: patients treated in 2015–2021 derived greater survival benefit from adjuvant therapy than those in 2007–2014; female patients experienced worse CSS with adjuvant therapy, which was identified as an independent risk factor; among patients receiving preoperative systemic therapy alone, continuing systemic therapy postoperatively showed superior outcomes compared with radiotherapy or combined treatment; and among patients receiving preoperative chemoradiotherapy, no significant differences were observed across different postoperative adjuvant strategies. The findings suggest that while postoperative adjuvant therapy does not provide an overall survival advantage, significant subgroup heterogeneity exists, indicating that clinical decisions should be individualized based on diagnosis year, gender, and preoperative treatment regimen.

**Supplementary Information:**

The online version contains supplementary material available at 10.1038/s41598-026-39930-5.

## Introduction

Esophageal cancer is a malignant tumor with persistently high global incidence and mortality, ranking seventh globally among causes of cancer-related deaths^1^.In cases of locally advanced esophageal cancer, surgery following neoadjuvant therapy has emerged as a globally accepted standard treatment approach, which improves surgical resection rates and survival outcomes by reducing preoperative tumor size and downstaging the disease^2–6^.

Nonetheless, the validity of postoperative adjuvant therapy (adjuvant systemic therapy, adjuvant radiotherapy, or their combination) in esophageal cancer patients who received neoadjuvant therapy before surgical intervention remains controversial. Available evidence has not reached a consensus on the survival advantage offered by postoperative adjuvant treatment. Some studies have suggested that postoperative adjuvant therapy can eradicate residual tumor cells, reduce recurrence risk, and improve survival^7–9^; others have indicated that adjuvant therapy fails to confer a survival benefit^10,11^ and may even deteriorate survival outcomes^12^. Most existing studies are limited by small sample sizes or single center designs, lack systematic analysis of large-scale real-world data, or only compare with the surgery-only group, making their conclusions difficult to extrapolate to the current clinical context of multimodal integrated therapy.

Given this context, this research sought to methodically evaluate the validity of postoperative adjuvant therapy in terms of survival advantage in esophageal cancer patients who had received neoadjuvant therapy prior to surgery, and to investigate subgroup variations in survival advantage by drawing on large-sample data retrieved from the Surveillance, Epidemiology, and End Results (SEER) database.

## Methods

### Patients

This population-based study utilized data obtained from the Surveillance, Epidemiology, and End Results (SEER) database. Esophageal cancer patients who had undergone neoadjuvant therapy before surgery were identified through the National Cancer Institute’s SEER*Stat software (version 8.4.5; note: corrected to official spelling) during the period from 2007 to 2021. Eligible cases were retrieved from the SEER-17 registry database (2000–2021) and fulfilled the following inclusion criteria: primary esophageal tumor, administration of neoadjuvant therapy (systemic therapy and/or radiotherapy) before surgery and undergoing esophagectomy. Patients were excluded if they had an unknown cause of death, incomplete baseline data, T0 stage, loss to follow-up before 2021, or survival time ≤ 2 months.

**Fig. 1:**
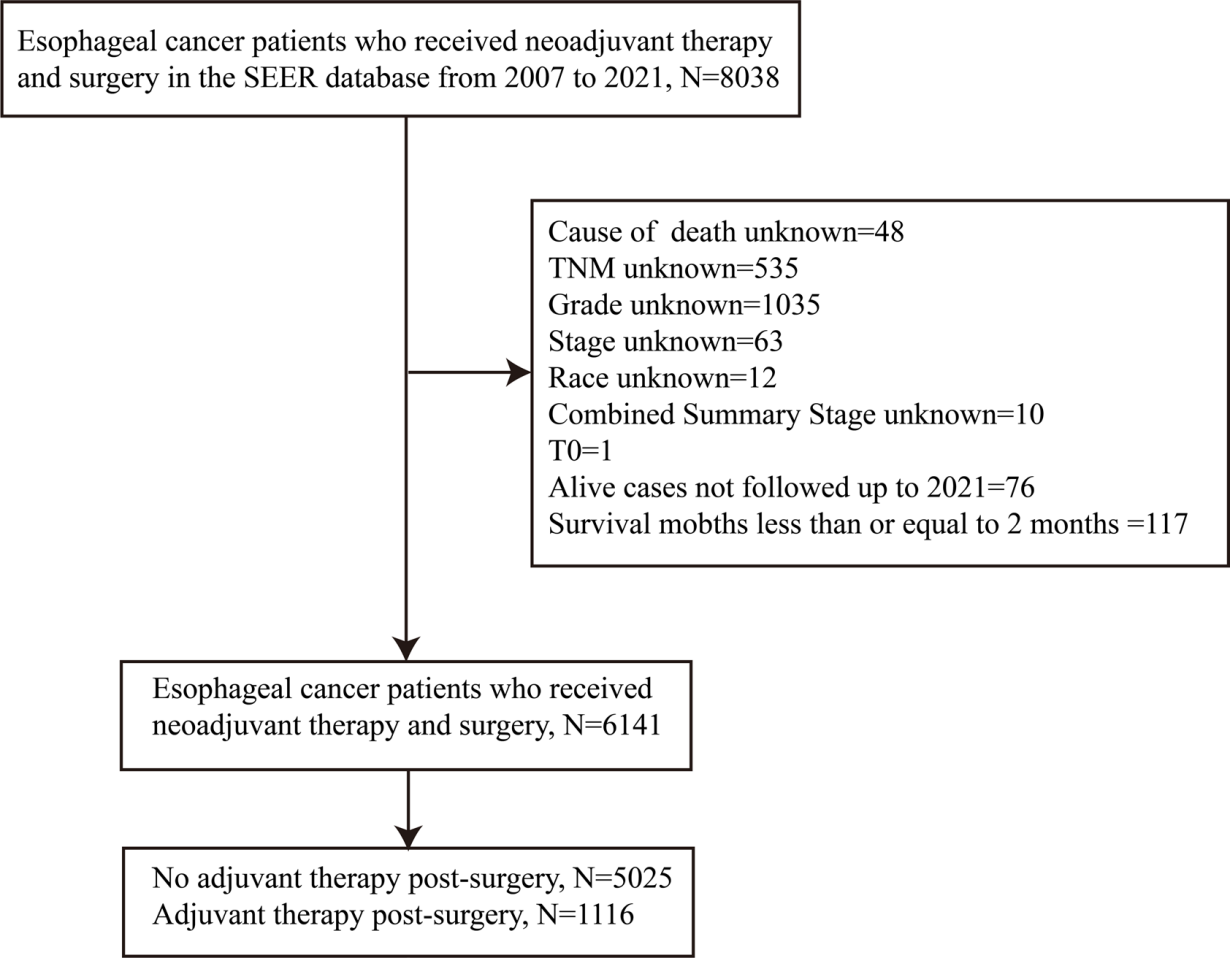
Inclusion and exclusion flow diagram for SEER esophageal cancer patients receiving neoadjuvant therapy and surgery, with or without adjuvant therapy from 2007 to 2021.

Patients given neoadjuvant therapy preoperatively and adjuvant therapy (systemic therapy and/or radiotherapy) postoperatively were categorized into the adjuvant therapy group; those who received neoadjuvant therapy preoperatively, but no adjuvant therapy postoperatively were assigned to the no adjuvant therapy group. Ultimately, the study enrolled a total of 6141 participants, comprising 1116 individuals assigned to the adjuvant therapy group and 5025 individuals allocated to the no adjuvant therapy group. (Fig. 1).

### Study variables

Variables for each patient were retrieved from the SEER database, including sex (female, male), age (20 to 85 + years), race (White, Black, and other), year of diagnosis (2007–2021), histologic type (adenocarcinoma, squamous cell carcinoma, other), primary site (lower third of esophagus, middle third of esophagus, other), combined summary stage(distant, localized, regional), number of primary tumors (single primary, 1st of two or more primaries, 2nd of two or more primaries, 3rd of three or more primaries), T stage, N stage, M stage, stage, grade, sequence of surgery and systemic therapy, sequence of surgery and radiotherapy, overall survival status, and cancer-specific survival status, survival months. T/N/M stage denotes SEER-derived AJCC staging integrating clinical and pathological data, with pathological findings prioritized. Grades denote esophageal cancer pathological differentiation: Grade I (well differentiated), Grade II (moderately differentiated), Grade III (poorly differentiated), and Grade IV (undifferentiated/anaplastic).

Some variables were reclassified as follows: age (< 60 years, ≥ 60 years); year of diagnosis (2007–2014, 2015–2021); and number of primary tumors (1, ≥ 2). Overall survival (OS) and cancer-specific survival (CSS) were defined as the primary endpoints.

### Statistical analysis

Comparisons of baseline clinical characteristics between the adjuvant therapy group and neoadjuvant therapy group, as well as the distribution of treatment modalities between the 2007–2014 and 2015–2021 cohorts, were conducted using the Chi-square test. Propensity score matching (PSM) with a 1:1 ratio, nearest neighbor as the method, and a caliper of 0.01 was applied to mitigate selection bias related to baseline variables. To identify independent prognostic factors tied to OS and CSS, univariate and multivariate Cox proportional hazards regression analyses were utilized, and findings were described as hazard ratios (HR) and 95% confidence intervals (CI). Survival curves were constructed via the Kaplan-Meier approach, and intergroup disparities in survival outcomes were assessed utilizing the log-rank test. All statistical analyses were executed with the IBM SPSS Statistics (version 26.0; IBM Corporation). A *P* < 0.05 threshold was set to denote statistical significance in this study.

## Results

### Patient characteristics

From the SEER database, 6141 esophageal cancer patients diagnosed between 2007 and 2021 were identified, with 1116 in the adjuvant therapy group and 5025 in the no adjuvant therapy group. The two groups differed in baseline characteristics such as gender, age, year of diagnosis, histologic type, primary site, and TNM stage (all *P* < 0.05). Specifically, the adjuvant therapy group exhibited a greater proportionate representation of males. males (87.1% vs. 83.5%), patients aged < 60 years (38.2% vs. 32.5%), adenocarcinoma (82.0% vs. 78.2%) (Table 1). After 1:1 PSM, 1,068 patients were included in each group, with all baseline characteristics well-balanced (all *P* > 0.05) and thus comparable (Supplemental Table 1).

**Table 1:** Baseline demographic and clinical characteristics of esophageal cancer patients with and without postoperative adjuvant therapy before propensity score matching.

	Adjuvant therapy (*N* = 1116)	No adjuvant therapy (*N* = 5025)	*P*
Sex, No. (%)			0.003
Female	144 (12.9%)	828 (16.5%)	
Male	972 (87.1%)	4197(83.5%)	
Age, No. (%)			**< 0.001**
< 60	426 (38.2%)	1634 (32.5%)	
≥ 60	690 (61.8%)	3391 (67.5%)	
Race, No. (%)			**0.035**
Black	41 (3.70%)	231 (4.6%)	
White	1008 (90.3%)	4572 (91.0%)	
Other	67 (6.0%)	222 (4.4%)	
Year of diagnosis, No. (%)			**< 0.001**
2007–2014	451 (40.4%)	2627 (52.3%)	
2015–2021	665 (59.6%)	2398 (47.7%)	
Histologic type ICD-O-3			**0.010**
Adenocarcinoma	915 (82.0%)	3932 (78.2%)	
Squamous cell carcinoma	171 (15.3%)	965 (19.2%)	
Other	30 (2.7%)	128 (2.5%)	
Primary Site-labeled			**0.031**
Lower third of esophagus	917 (82.2%)	3952 (78.6%)	
Middle third of esophagus	102 (9.1%)	543 (10.8%)	
Other	97 (8.7%)	530 (10.5%)	
Combined summary stage			**< 0.001**
Distant	182 (16.3%)	582 (11.6%)	
Localized	129 (11.6%)	839 (16.7%)	
Regional	805 (72.1%)	3604 (71.7%)	
Sequence number			0.306
One primary only	871(78.0%)	3850(76.6%)	
≥ 2 primaries	245(22.0%)	1175(23.4%)	
T			**0.019**
T1	130 (11.6%)	647 (12.9%)	
T2	167 (15.0%)	883 (17.6%)	
T3	747 (66.9%)	3251 (64.7%)	
T4	72 (6.5%)	244 (4.9%)	
N			**< 0.001**
N0	295 (26.4%)	1814 (36.1%)	
N1	664 (59.5%)	2895(57.6%)	
N2	111 (9.9%)	246 (4.9%)	
N3	46 (4.1%)	70 (1.4%)	
M			**< 0.001**
M0	1008 (90.3%)	4693 (93.4%)	
M1	108 (9.7%)	332 (6.6%)	
Stage			**< 0.001**
I	63(5.6%)	391(7.8%)	
II	287(25.7%)	1784(35.5%)	
III	620(55.6%)	2476(49.3%)	
IV	146(13.1%)	374(7.4%)	
Grade			0.190
Grade I	51 (4.6%)	246 (4.9%)	
Grade II	469 (42.0%)	2259 (45.0%)	
Grade III	584 (52.3%)	2474 (49.2%)	
Grade IV	12 (1.1%)	46 (0.9%)	

### Overall impact of postoperative adjuvant therapy

**Fig. 2:**
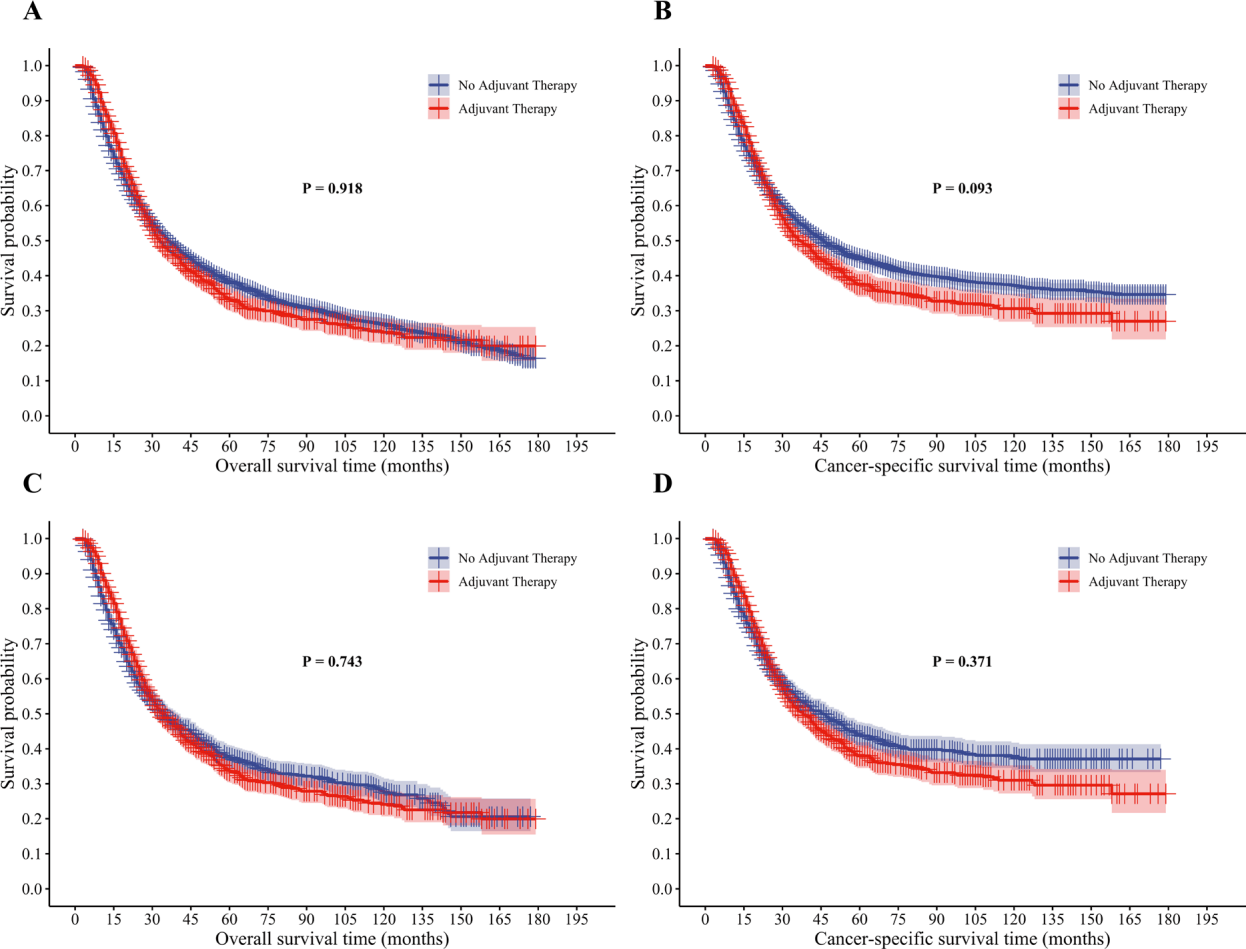
Overall and cancer-specific survival analysis of esophageal cancer patients before and after PSM. (**A**) Overall survival analysis before PSM; (**B**) Cancer-specific survival analysis before PSM; (**C**) Overall survival analysis after PSM; (**D**) Cancer-specific survival analysis after PSM.

Before PSM, between the adjuvant therapy group and the no adjuvant therapy group, no statistical differences in OS or CSS were found (OS: *P* = 0.918; CSS: *P* = 0.093). Following PSM, the survival differences between the two groups remained non-significant (OS: *P* = 0.743; CSS: *P* = 0.371). The survival curves exhibited a “time-dependent crossover” pattern, specifically a phenomenon where the adjuvant therapy group showed better early survival, with a subsequent reversal favoring the no adjuvant therapy group in the late phase. The reversal occurred between 25 and 35 months; however, this crossover trend approached statistical significance only in the pre-PSM CSS analysis (*P* = 0.093), while the remaining analyses (pre-PSM OS, post-PSM OS, and post-PSM CSS) did not reach statistical significance (Fig. 2A–D).

Pre-PSM multivariate Cox regression models revealed that patients diagnosed between 2015 and 2021 and those with two or more primary tumors were protective factors for both OS and CSS relative to the reference factor. In contrast, N1, N2, and N3 stages emerged as independent hazard factors that exert an influence on both OS and CSS in comparison with N0. Post-PSM multivariate Cox regression models demonstrated that a diagnosis between 2015 and 2021 was an independent protective prognostic factor for OS and CSS, while Grade IV was an independent hazard factor for both outcomes. Both pre- and post-PSM Cox regression models confirmed that adjuvant therapy intervention failed to qualify as an independent prognostic factor for OS or CSS (Supplemental Tables 2, 3).

### Subgroup analysis results

The specific combinations of preoperative neoadjuvant and postoperative adjuvant systemic therapy and radiotherapy in the adjuvant and no adjuvant therapy groups, along with differences in treatment modality distribution between the 2007–2014 and 2015–2021 subgroups, are detailed in the supplementary material (Supplemental Table 4).In the no adjuvant therapy group, the proportion of patients receiving neoadjuvant systemic therapy alone (6.47% vs. 3.53%, *P* < 0.001) and neoadjuvant systemic therapy plus radiotherapy (78.10% vs. 74.28%, *P* < 0.001) differed significantly between the two periods. In the adjuvant therapy group, the proportion of patients receiving adjuvant radiotherapy after neoadjuvant systemic therapy (2.05% vs. 0.95%, *P* < 0.001) and adjuvant systemic therapy after neoadjuvant systemic therapy plus radiotherapy (5.59% vs. 13.16%, *P* < 0.001) also showed significant differences. No significant differences were observed in the distribution of other treatment modalities between the two periods.

**Fig. 3:**
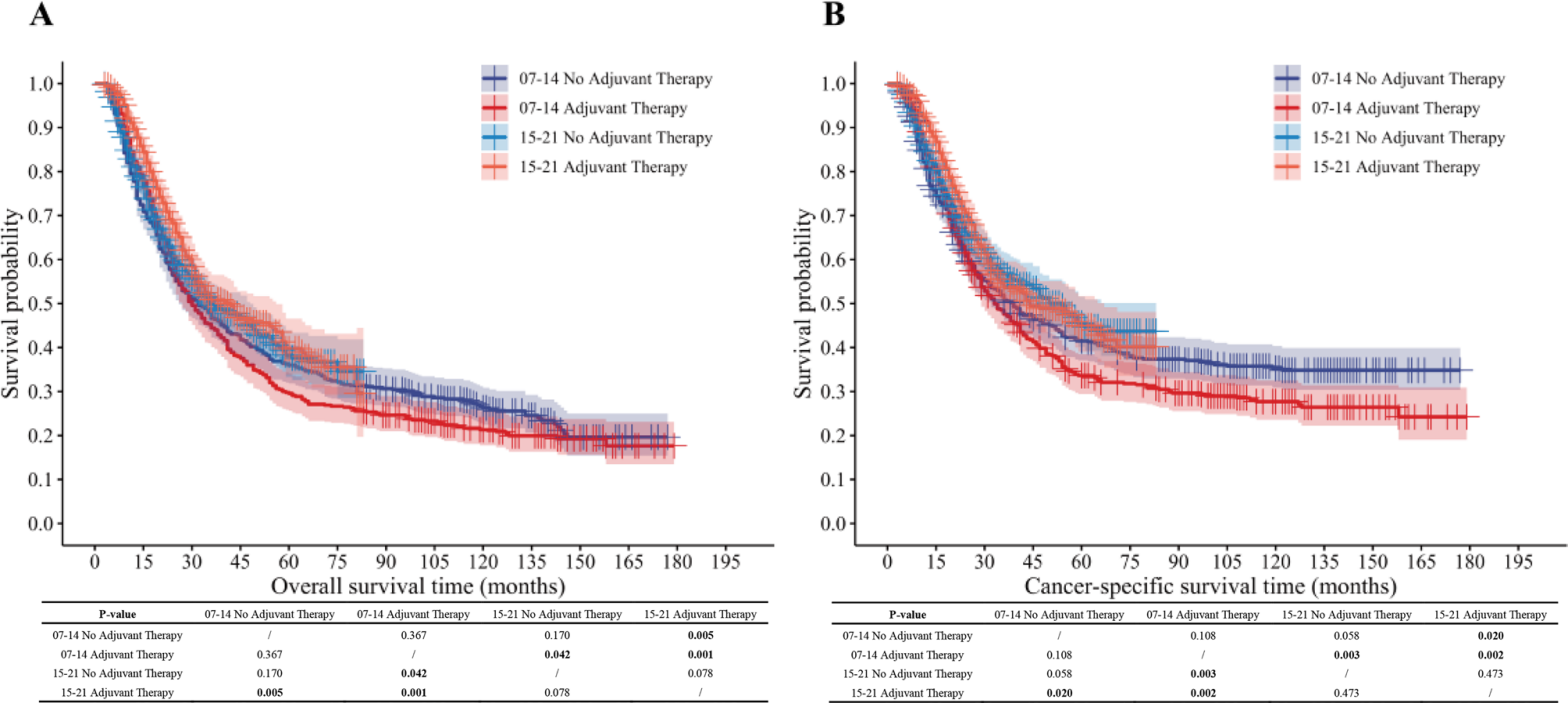
Overall and cancer-specific survival analysis of patients stratified by year of diagnosis and adjuvant therapy. (**A**) Overall survival analysis; (**B**) Cancer-specific survival analysis.

Subgroup analyses stratified by year of diagnosis showed no survival benefit from adjuvant therapy within either time period (2007–2014 or 2015–2021; all *P* > 0.05). When comparing across time periods, the survival benefit of adjuvant therapy was greater in 2015–2021 than in 2007–2014 (OS: *P* = 0.001; CSS: *P* = 0.002), whereas no statistical difference in the survival benefit derived from neoadjuvant therapy was detected between these two periods. Notably, the survival benefit of adjuvant therapy in 2015–2021 was superior to that of neoadjuvant therapy in 2007–2014 (OS: *P* = 0.005; CSS: *P* = 0.020) (Fig. 3A, B).

Gender-stratified subgroup analysis demonstrated that among female patients, a statistical difference in cancer-specific survival (CSS) existed between two groups (*P* = 0.027) (Table 2).Multivariate Cox regression analysis revealed that further postoperative adjuvant therapy was an independent risk factor for survival in female patients with esophageal cancer who underwent neoadjuvant therapy followed by surgery (HR  1.582, 95% CI 1.044–2.397, *P* = 0.031) (Table 3).

**Table 2:** Subgroup analyses of overall survival (OS) and cancer-specific survival (CSS) after propensity score matching according to different clinicopathological factor. Survival curves were compared using the log-rank test with pairwise comparisons performed between each stratum.

	Adjuvant Therapy (*N* = 1068)	No Adjuvant Therapy (*N* = 1068)	OS *P*	CSS *P*
Sex, No. (%)				
Female	138(12.9%)	125(11.7%)	0.108	**0.027**
Male	930(87.1%)	943(88.3%)	0.346	0.882
Age, No. (%)				
< 60	398(37.3%)	384(36.0%)	0.802	0.616
≥ 60	670(62.7%)	684(64.0%)	0.535	0.479
Race				
Black	39(3.7%)	30(2.8%)	0.669	0.733
White	972(91.0%)	996(93.3%)	0.707	0.453
Other	57(5.3%)	42(3.9%)	0.669	0.240
Year of diagnosis				
2007–2014	450(42.1%)	450(42.1%)	0.367	0.108
2015–2021	618(57.9%)	618(57.9%)	0.078	0.473
Histologic type ICD-O-3				
Adenocarcinoma	874(81.8%)	896(83.9%)	0.585	0.599
Squamous cell carcinoma	167(15.6%)	148(13.9%)	0.801	0.302
Other	27(2.5%)	24(2.2%)	0.793	0.851
Primary Site-labeled				
Lower third of esophagus	881(82.5%)	894(83.7%)	0.609	0.621
Middle third of esophagus	96(9.0%)	88(8.2%)	0.793	0.338
Other	91(8.5%)	86(8.1%)	0.882	0.603
Combined summary stage				
Distant	166(15.5%)	161(15.1%)	0.945	0.547
Localized	129(12.1%)	132(12.4%)	0.128	0.111
Regional	773(72.4%)	775(72.6%)	0.270	0.997
Sequence number				
One primary only	831(77.8%)	836(78.3%)	0.743	0.555
≥ 2 primaries	237(22.2%)	232(21.7%)	0.996	0.369
T				
T1	124(11.6%)	123(11.5%)	0.895	0.478
T2	162(15.2%)	157(14.7%)	0.592	0.646
T3	719(67.3%)	732(68.5%)	0.829	0.385
T4	63(5.9%)	56(5.2%)	0.753	0.706
N				
N0	294(27.5%)	297(27.8%)	0.582	0.166
N1	660(61.8%)	657(61.5%)	0.870	0.383
N2	91(8.5%)	93(8.7%)	0.134	0.132
N3	23(2.2%)	21(2.0%)	0.717	0.655
M				
M0	968(90.6%)	969(90.7%)	0.914	0.228
M1	100(9.4%)	99(9.3%)	0.400	0.394
Stage				
I	63(5.9%)	64(6.0%)	0.252	0.203
II	287(26.9%)	272(25.5%)	0.803	0.398
III	605(56.6%)	620(58.1%)	0.871	0.341
IV	113(10.6%)	112(10.5%)	0.183	0.199
Grade				
Grade I	49(4.6%)	51(4.8%)	0.597	0.963
Grade II	458(42.9%)	450(42.1%)	0.700	0.306
Grade III	550(51.5%)	558(52.2%)	0.492	0.819
Grade IV	11(1.0%)	9(0.8%)	0.624	0.432

**Table 3:** Multivariable cox proportional hazards regression analysis of cancer-specific survival in female patients.

	HRs(95% CI)	*P*
Race		
Black	Reference	
White	0.441(0.233–0.834)	**0.012**
Other	0.792(0.311–2.015)	0.624
T		
T1	Reference	
T2	0.386(0.154–0.966)	**0.042**
T3	0.839(0.430–1.636)	0.607
T4	0.958(0.324–2.826)	0.937
Therapy		
No adjuvant therapy	Reference	
Adjuvant therapy	1.582(1.044–2.397)	**0.031**

Hazard ratios (HRs) and 95% confidence intervals (CIs) were estimated using Cox proportional hazards regression models.

**Fig. 4:**
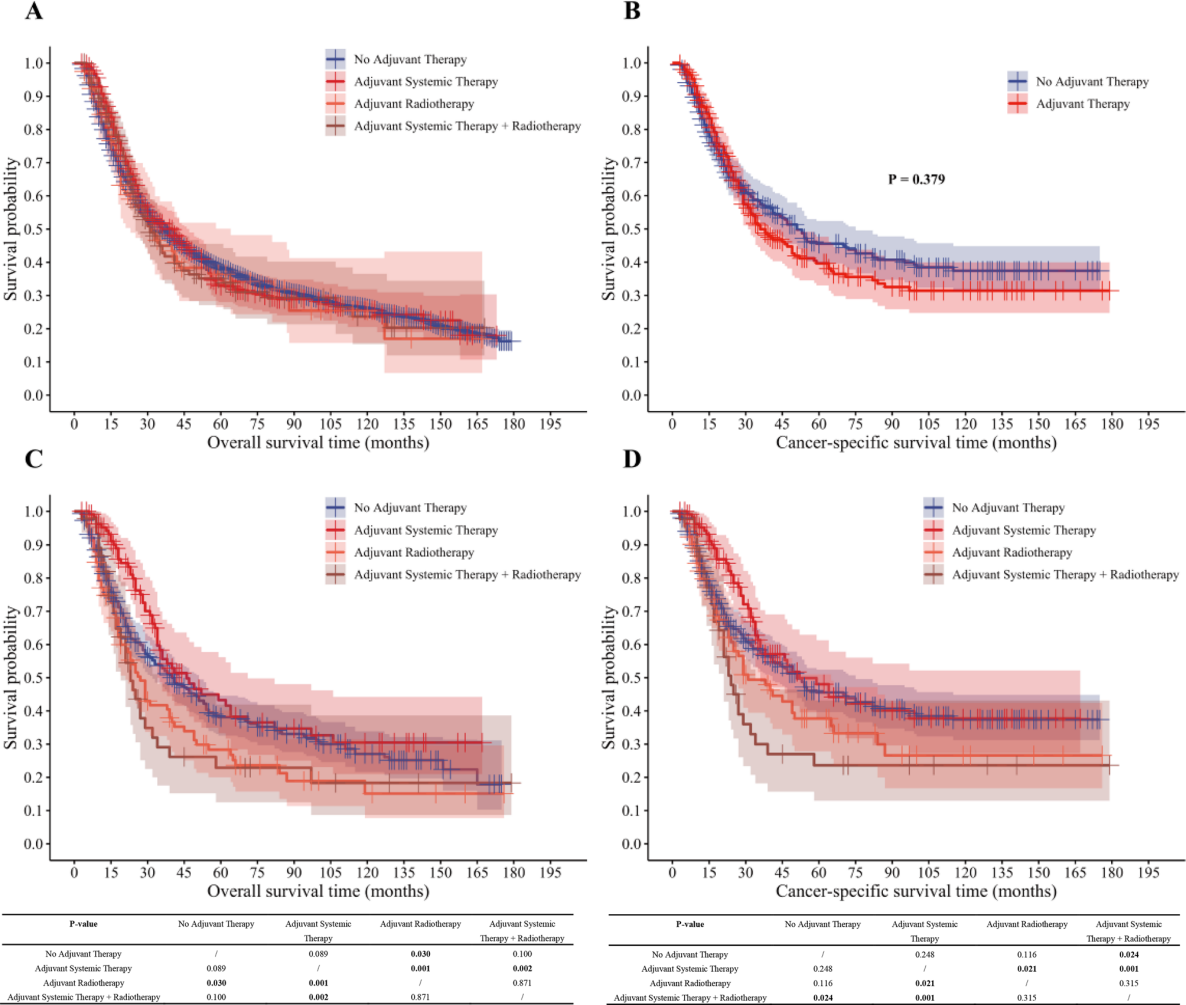
Overall and cancer-specific survival analysis of esophageal cancer patients treated with neoadjuvant systemic therapy alone. (**A**) Overall survival analysis; (**B**) Cancer-specific survival analysis; (**C**) Kaplan–Meier OS curve by adjuvant therapy regimen; (**D**) Kaplan–Meier CSS curve by adjuvant therapy regimen.

Subgroup analyses of different adjuvant treatment regimens were performed with neoadjuvant regimens standardized across groups. Among patients who received preoperative systemic therapy alone, no statistical variation in the efficacy of survival outcomes was detected between adjuvant therapy and no adjuvant therapy (Fig. 4A, B). Further stratification by adjuvant regimens showed that, in patients with preoperative systemic therapy alone, postoperative adjuvant systemic therapy differed from adjuvant radiotherapy and from adjuvant combination therapy in terms of OS (*P* = 0.001, *P* = 0.002) and CSS (*P* = 0.021, *P* = 0.001). Additionally, individuals who lacked postoperative adjuvant therapy exhibited superior OS compared with those undergoing postoperative radiotherapy (*P* = 0.030) and demonstrated superior CSS compared with those undergoing postoperative combination therapy (*P* = 0.024).

No statistical variation in terms of survival benefit emerged when comparing the postoperative adjuvant systemic therapy group with the no adjuvant therapy group. However, adjuvant systemic therapy appeared to confer a survival benefit up to approximately 30 months (Fig. 4C, D). The baseline characteristics of the four adjuvant treatment subgroups corresponding to Fig. 4 are presented in the supplementary material (Supplemental Table 5).

**Fig. 5:**
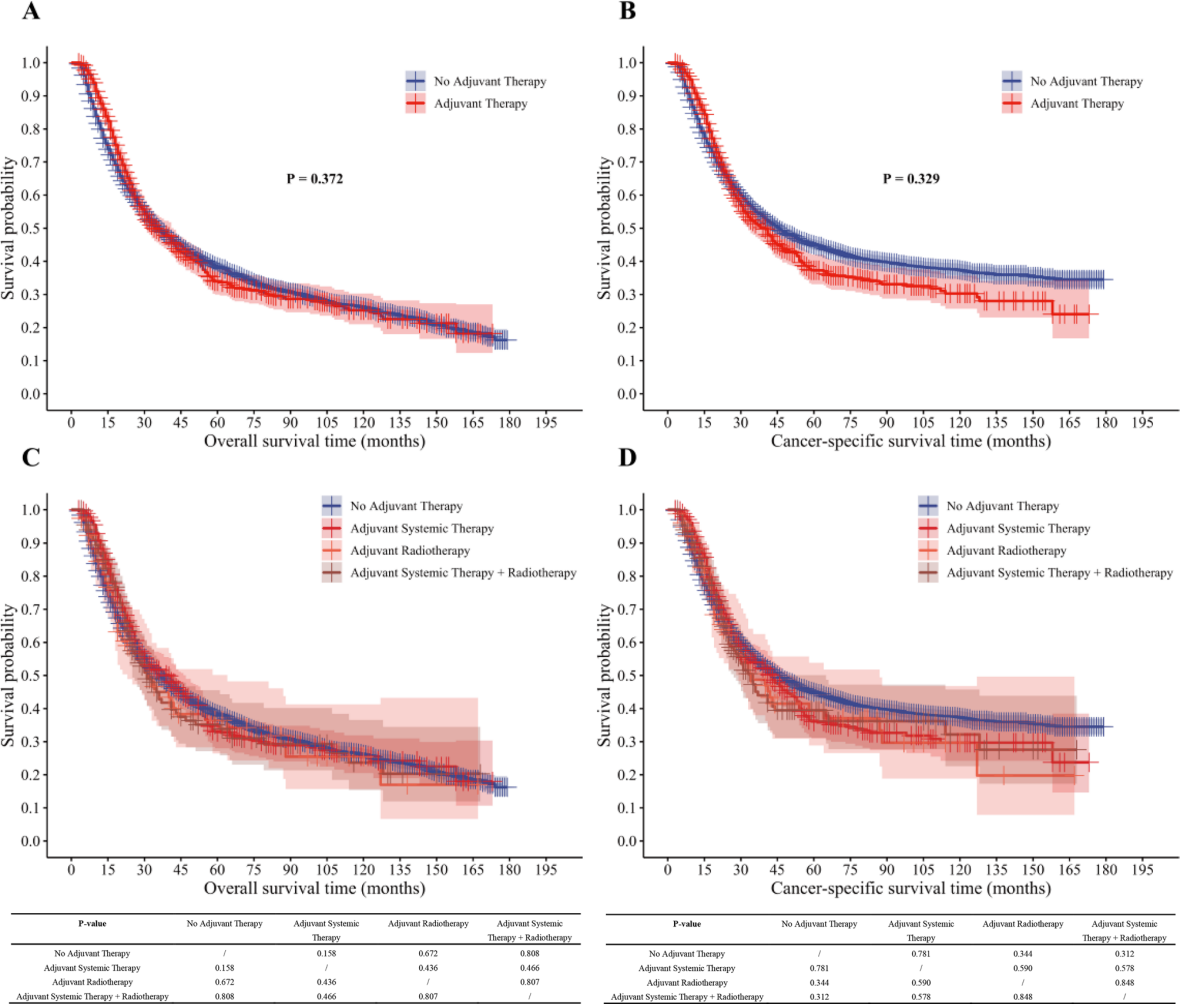
Overall and cancer-specific survival analysis of esophageal cancer patients treated with neoadjuvant systemic therapy + Radiotherapy. (**A**) Overall survival analysis; (**B**) Cancer-specific survival analysis; (**C**) Kaplan–Meier OS curve by adjuvant therapy regimen; (**D**) Kaplan–Meier CSS curve by adjuvant therapy regimen.

In patients who undergoing preoperative systemic therapy plus radiotherapy, regardless of whether adjuvant therapy was administered, or the specific type of adjuvant regimen used, no statistical differences in postoperative survival outcomes were detected (Fig. 5A–D).

## Discussion

This study conducted a systematic assessment of how postoperative adjuvant therapy influences survival outcomes of esophageal cancer patients who had undergone preoperative neoadjuvant therapy, using the SEER database as data source.

Data from this investigation reveals that esophageal cancer patients derive no survival benefit from postoperative adjuvant therapy, as the treatment failed to improve OS or CSS in the overall population either before or after PSM. In a retrospective clinical study, individuals undergoing neoadjuvant therapy prior to surgery achieved an R0 resection rate of 92%, whereas the rate stood at 69% in the group treated with surgery alone (*P* < 0.001)^5^. It is suggested that neoadjuvant therapy may achieve a “treatment saturation effect” through mechanisms such as tumor downstaging and eradication of micrometastases, thereby limiting the incremental value of postoperative adjuvant therapy.

A previous network meta-analysis further supports that neoadjuvant therapy prior to surgery provides a survival benefit superior to surgery alone, whereas surgery followed by adjuvant therapy fails to show such a benefit^13^. These findings reinforce the central role of neoadjuvant therapy in the multimodal management of esophageal cancer and highlight the limitations in therapeutic efficacy of postoperative adjuvant intervention. Given that neoadjuvant therapy has adequately controlled tumor progression, postoperative adjuvant therapy fails to further prolong survival, and its clinical utility warrants re-evaluation.

Notably, the survival curves exhibited a “time-dependent crossover”, the adjuvant therapy group showed a slight early survival advantage, which reversed in the later period with the no adjuvant therapy group performing better, with reversal points concentrated between 25 and 35 months. This indicates that the influence of adjuvant therapy on survival is dynamically variable. This phenomenon may be related to selection bias in the study population. In clinical practice, successful delivery of adjuvant therapy depends on adequate patient physical performance, certain individuals in the no adjuvant therapy group may have foregone postoperative adjuvant therapy due to reduced physical function following neoadjuvant therapy and surgery^14^, rendering them unable to tolerate further treatment. Given the shorter survival expectancy of this subgroup, the no adjuvant therapy group appears disadvantaged in short-term survival. Additionally, cumulative toxicity may be a potential mechanism. Although neoadjuvant therapy achieves tumor downstaging and clearance of micrometastases, a small number of residual tumor foci may remain. Postoperative adjuvant therapy may further reduce tumor burden in the short term^15^, accounting for the early advantage in the survival curve. This early benefit may involve cumulative or delayed toxicity of adjuvant therapy^4,16^, where toxic effects manifest after initial antitumor effects, gradually offsetting the early advantage. A “time-dependent crossover” in survival curves occurs when long-term toxic risks outweigh the early survival benefit from antitumor therapy.

Temporal shifts in esophageal cancer treatment modalities are observed, which may reflect the paradigm transition in clinical management around 2015. One notable trend is the increased adoption of combined therapy regimens, which appears consistent with the broader clinical uptake of the CROSS^17^neoadjuvant protocol after its long-term efficacy was validated. This protocol has gradually become a standard preoperative approach for locally advanced esophageal cancer, contributing to the integration of multimodal strategies in routine practice. Meanwhile, the adjuvant therapy cohort showed a higher proportion of postoperative systemic therapy, potentially related to the introduction of more effective systemic agents in recent years. It is also worth noting that most other treatment modalities remained relatively stable over the study period, with no significant temporal differences observed. This stability may reflect the established role of these regimens in clinical practice, which has not undergone major adjustments during the study timeframe.

In the year-based subgroup analysis, for the same treatment modality, survival differences between two groups became statistically significant with increasing years, whereas no observed statistical disparities were evident amongst the two groups over the same timeframe. The gradual improvement in the survival benefit of postoperative adjuvant therapy is closely associated with pivotal clinical advances that collectively underpin the overall progress of medical technology^18^, which mainly include enhanced perioperative management protocols, innovative radiotherapy technologies, and the emergence of adjuvant immunotherapy. First, perioperative management has been optimized through Enhanced Recovery After Surgery (ERAS) protocols, which have decreased postoperative complications and facilitated earlier initiation and completion of adjuvant therapy^19^. Second, significant advances in radiotherapy technology—especially the widespread application of intensity-modulated radiotherapy (IMRT)—have substantially reduced treatment-related toxicities compared with three-dimensional conformal radiotherapy (3D-CRT)^20,21^, which has allowed more patients to complete postoperative chemoradiotherapy with better tolerance. In addition, contemporary observational studies have reported improved overall survival with adjuvant chemoradiotherapy or chemotherapy in the modern IMRT era^22,23^, further supporting that postoperative treatments delivered in later years are more effective and better tolerated. Third, the therapeutic landscape after esophagectomy has expanded markedly in recent years. The introduction of adjuvant immunotherapy, most notably nivolumab as demonstrated in the CheckMate-577 trial, represents a paradigm shift and provides meaningful survival benefits in patients with residual disease after neoadjuvant chemoradiotherapy^24^. These favorable clinical outcomes and relative advantages, however, warrant further validation through extended follow-up periods and more granular stratified analyses.

Gender subgroup analysis results indicated that adjuvant therapy was an independent hazard factor for CSS in female esophageal cancer patients. For the gender differences in survival outcomes associated with adjuvant therapy, the underlying mechanisms may stem from the following aspects. First, female patients face an elevated risk of severe adverse events when receiving adjuvant therapy^25^. This may directly affect treatment tolerance and subsequent quality of survival. Second, women’s drug clearance is lower than men’s, which can lead to more toxic accumulation^26^and elevate the hazard of cancer-related death. Besides, given the role of gut microbiota in regulating metabolic and immunoinflammatory pathways, gut microbiota may also be relevant^27^. Therefore, clarifying such gender differences is of important clinical significance for optimizing therapeutic decisions in esophageal cancer, suggesting that individualized risk-benefit assessment is needed when applying adjuvant therapy in female esophageal cancer patients.

In this study, stratified analyses showed that survival outcomes in esophageal cancer patients differed significantly between different combinations of neoadjuvant and postoperative adjuvant regimens.

Among patients who received preoperative systemic therapy alone, postoperative adjuvant systemic therapy was associated with better OS and CSS than postoperative adjuvant radiotherapy or adjuvant systemic therapy combined with radiotherapy (*P* < 0.05). It also conferred a clear survival advantage over the no adjuvant therapy group, particularly in the early stage (within approximately 30 months). First, this finding confirms the efficacy of adjuvant systemic therapy in patients who received neoadjuvant therapy followed by surgery, which is consistent with results from a small-sample retrospective study^28^, despite limited current evidence supporting a survival benefit of postoperative adjuvant systemic therapy in esophageal cancer^29^. Second, it suggests that postoperative adjuvant radiotherapy may be of limited value. As a local treatment modality, postoperative radiotherapy alone primarily targets residual lesions in the surgical field but has limited efficacy against systemic micrometastases. Postoperative radiotherapy for esophageal cancer often involves the thoracic and mediastinal regions, with radiotherapy-induced cardiorespiratory toxicity linked to poorer survival outcomes^30,31^. Thus, postoperative adjuvant radiotherapy may have limited efficacy in controlling micrometastases and preventing recurrence while increasing the risk of serious treatment-related comorbidities. Additionally, toxicity from postoperative adjuvant systemic therapy combined with radiotherapy may offset the survival benefits of systemic therapy alone, resulting in inferior outcomes compared with postoperative adjuvant systemic therapy alone.

Among patients who received preoperative systemic therapy plus radiotherapy, no statistical disparities were identified in either OS or CSS across the four subgroups stratified by postoperative adjuvant strategies: systemic therapy alone, radiotherapy alone, systemic therapy combined with radiotherapy, or no adjuvant treatment. The efficacy of preoperative systemic therapy plus radiotherapy has been validated in multiple prior studies and is currently the preferred neoadjuvant regimen^5,6,29^. This combination was also the most used neoadjuvant regimen in our study. Notably, the results of this subgroup exhibited a substantial degree of congruence with the aggregated analysis of the total population, further validating the “treatment saturation effect.” Together with the outcomes of the subgroup analysis where neoadjuvant therapy was systemic therapy alone, this implies that the intensive neoadjuvant regimen of systemic therapy plus radiotherapy may achieve superior local control and systemic effects^4^, reducing the proliferative potential and metastatic risk of residual lesions to a lower level. At this point, additional postoperative adjuvant therapy (or its absence) has a limited impact on survival outcomes.

In summary, the results of this study establish a framework for the stratified administration of multifaceted therapy in esophageal cancer, and routine application of adjuvant therapy after neoadjuvant therapy prior to surgery is not recommended. Specifically, among patients who received preoperative systemic therapy plus radiotherapy, no statistical difference in survival benefit was observed between postoperative adjuvant therapy group and no adjuvant therapy group. Routine administration of adjuvant therapy is thus unnecessary to avoid the toxic burden of overtreatment. For patients who received preoperative systemic therapy alone, despite a trend toward better survival with postoperative adjuvant systemic therapy compared with other adjuvant regimens, no statistical difference in survival benefit was noted versus no adjuvant therapy. Furthermore, the risk of postoperative adjuvant therapy in female patients warrants attention, and clinical decisions should be made cautiously, considering their biological characteristics and toxicity tolerance.

### Limitations

Several limitations are inherent to the present research. First, critical information including margin status and comorbidities is not available in the SEER database, which may compromise the accuracy of the association between adjuvant therapy and survival outcomes. Second, the database does not include parameters such as systemic treatment duration and radiotherapy dose, precluding further exploration of dose-response relationships. Third, given the long duration of the study period, treatment modalities underwent modest yet unavoidable shifts over time; while we employed temporal stratification and PSM to mitigate this confounding effect, residual biases related to changes in treatment practices may still persist. Furthermore, the proportion of female patients within the subgroup sample is comparatively minimal, thereby necessitating the validation of these results through external cohorts.

## Conclusion

In esophageal cancer patients treated with neoadjuvant therapy prior to surgery, postoperative adjuvant therapy failed to yield an enhancement in OS or CSS in the overall population. However, survival outcomes exhibit subgroup heterogeneity, highlighting the need for individualized decision-making in clinical practice that integrates characteristics such as year of diagnosis, gender, and preoperative treatment regimen to avoid the indiscriminate use of adjuvant therapy. Using large-sample real-world data, this study clarifies reference boundaries for the application of adjuvant therapy, providing a basis for optimizing multimodal treatment strategies and facilitating the transition of esophageal cancer management from “standardization” to “precision.”

## Supplementary Information

Below is the link to the electronic supplementary material.

Supplementary Material 1

## Data Availability

The datasets generated during and/or analyzed during the current study are available in the Surveillance, Epidemiology, and End Results (SEER) repository.
