# Supplementary material for: Impact of adjuvant therapy on survival in esophageal cancer patients after neoadjuvant therapy investigated by a population based cohort study
Source: Sci Rep. 2026 Apr 13;16:17215. doi: 10.1038/s41598-026-39930-5 (PMC13234424; doi:10.1038/s41598-026-39930-5)
Supplement: Supplementary file 1 — Supplementary Material 1 [file 41598_2026_39930_MOESM1_ESM.docx]

**Impact of Adjuvant Therapy on Survival in Esophageal Cancer Patients After Neoadjuvant Therapy Investigated by a Population Based Cohort Study**

**Supplementary information**

|  | Adjuvant Therapy  (N=1068) | No Adjuvant Therapy  (N=1068) | P |
| --- | --- | --- | --- |
| Sex, No. (%) |  |  | 0.392 |
| Female | 138(12.9%) | 125(11.7%) |  |
| Male | 930(87.1%) | 943(88.3%) |  |
| Age, No. (%) |  |  | 0.529 |
| <60 | 398(37.3%) | 384(36.0%) |  |
| ≥60 | 670(62.7%) | 684(64.0%) |  |
| Race |  |  | 0.154 |
| Black | 39(3.7%) | 30(2.8%) |  |
| White | 972(91.0%) | 996(93.3%) |  |
| Other | 57(5.3%) | 42(3.9%) |  |
| Year of diagnosis |  |  | 1.000 |
| 2007-2014 | 450(42.1%) | 450(42.1%) |  |
| 2015-2021 | 618(57.9%) | 618(57.9%) |  |
| Histologic Type ICD-O-3 |  |  | 0.450 |
| Adenocarcinoma | 874(81.8%) | 896(83.9%) |  |
| Squamous cell carcinoma | 167(15.6%) | 148(13.9%) |  |
| Other | 27(2.5%) | 24(2.2%) |  |
| Primary Site-labeled |  |  | 0.747 |
| Lower third of esophagus | 881(82.5%) | 894(83.7%) |  |
| Middle third of esophagus | 96(9.0%) | 88(8.2%) |  |
| Other | 91(8.5%) | 86(8.1%) |  |
| Combined Summary Stage |  |  | 0.945 |
| Distant | 166(15.5%) | 161(15.1%) |  |
| Localized | 129(12.1%) | 132(12.4%) |  |
| Regional | 773(72.4%) | 775(72.6%) |  |
| Sequence number |  |  | 0.794 |
| One primary only | 831(77.8%) | 836(78.3%) |  |
| ≥2 primaries | 237(22.2%) | 232(21.7%) |  |
| T |  |  | 0.894 |
| T1 | 124(11.6%) | 123(11.5%) |  |
| T2 | 162(15.2%) | 157(14.7%) |  |
| T3 | 719(67.3%) | 732(68.5%) |  |
| T4 | 63(5.9%) | 56(5.2%) |  |
| N |  |  | 0.987 |
| N0 | 294(27.5%) | 297(27.8%) |  |
| N1 | 660(61.8%) | 657(61.5%) |  |
| N2 | 91(8.5%) | 93(8.7%) |  |
| N3 | 23(2.2%) | 21(2.0%) |  |
| M |  |  | 0.941 |
| M0 | 968(90.6%) | 969(90.7%) |  |
| M1 | 100(9.4%) | 99(9.3%) |  |
| Stage |  |  | 0.897 |
| I | 63(5.9%) | 64(6.0%) |  |
| II | 287(26.9%) | 272(25.5%) |  |
| III | 605(56.6%) | 620(58.1%) |  |
| IV | 113(10.6%) | 112(10.5%) |  |
| Grade |  |  | 0.947 |
| Grade I | 49(4.6%) | 51(4.8%) |  |
| Grade II | 458(42.9%) | 450(42.1%) |  |
| Grade III | 550(51.5%) | 558(52.2%) |  |
| Grade IV | 11(1.0%) | 9(0.8%) |  |

Supplemental table 1 Presentation of propensity score matched (PSM) data based on baseline characteristics in esophageal cancer patients who received neoadjuvant therapy, stratified by postoperative adjuvant therapy group and no adjuvant therapy group.

|  | OS(N=6141) | | | | CSS(N=6141) | | | |
| --- | --- | --- | --- | --- | --- | --- | --- | --- |
|  | Univariate | | Multivariate | | Univariate | | Multivariate | |
| Characteristics | HR（95%CI） | P | HR（95%CI） | P | HR（95%CI） | P | HR（95%CI） | P |
| Sex |  |  |  |  |  |  |  |  |
| Female | Reference |  | Reference |  | Reference |  | Reference |  |
| Male | 1.280(1.165-1.406) | **<0.001** | 1.244(1.130-1.370) | **<0.001** | 1.321(1.188-1.468) | **<0.001** | 1.257(1.128-1.400) | **<0.001** |
| Age |  |  |  |  |  |  | Not selected |  |
| <60 | Reference |  | Reference |  | Reference |  |  |  |
| ≥60 | 1.099(1.026-1.176) | **0.007** | 1.162(1.084-1.245) | **<0.001** | 1.003(0.931-1.081) | 0.931 |  |  |
| Race |  |  | Not selected |  |  |  | Not selected |  |
| Black | Reference |  |  |  | Reference |  |  |  |
| White | 0.973(0.833-1.136) | 0.728 |  |  | 1.056(0.884-1.262) | 0.548 |  |  |
| Other | 0.963(0.778-1.192) | 0.731 |  |  | 1.102(0.869-1.398) | 0.422 |  |  |
| Year of diagnosis |  |  |  |  |  |  |  |  |
| 2007-2014 | Reference |  | Reference |  | Reference |  | Reference |  |
| 2015-2021 | 0.881(0.823-0.944) | **<0.001** | 0.828(0.768-0.893) | **<0.001** | 0.850(0.789-0.916) | **<0.001** | 0.796(0.733-0.864) | **<0.001** |
| Histologic Type ICD-O-3 |  |  | Not selected |  |  |  | Not selected |  |
| Adenocarcinoma | Reference |  |  |  | Reference |  |  |  |
| Squamous cell carcinoma | 0.980(0.902-1.066) | 0.641 |  |  | 0.923(0.840-1.015) | 0.097 |  |  |
| Other | 1.115(0.926-1.343) | 0.249 |  |  | 1.179(0.965-1.440) | 0.107 |  |  |
| Primary Site-labeled |  |  |  |  |  |  |  |  |
| Lower third of esophagus | Reference |  | Reference |  | Reference |  | Reference |  |
| Middle third of esophagus | 1.018(0.915-1.132) | 0.744 | 1.123(1.006-1.253) | **0.038** | 0.989(0.878-1.114) | 0.853 | 1.122(0.993-1.268) | 0.064 |
| Other | 1.317(1.189-1.458) | **<0.001** | 1.370(1.236-1.518) | **<0.001** | 1.242(1.107-1.393) | **<0.001** | 1.306(1.163-1.466) | **<0.001** |
| Combined Summary Stage |  |  |  |  |  |  |  |  |
| Distant | Reference |  | Reference |  | Reference |  | Reference |  |
| Localized | 0.494(0.438-0.558) | **<0.001** | 0.845(0.676-1.056) | 0.139 | 0.439(0.383-0.502) | **<0.001** | 0.861(0.672-1.102) | 0.235 |
| Regional | 0.692(0.633-0.758) | **<0.001** | 0.850(0.739-0.978) | **0.023** | 0.650(0.590-0.717) | **<0.001** | 0.849(0.729-0.988) | **0.035** |
| Sequence number |  |  |  |  |  |  |  |  |
| One primary only | Reference |  | Reference |  | Reference |  | Reference |  |
| ≥2 primaries | 0.841(0.780,0.908) | **<0.001** | 0.847(0.785-0.915) | <0.001 | 0.696(0.637-0.761) | **<0.001** | 0.722(0.660-0.790) | **<0.001** |
| T |  |  |  |  |  |  |  |  |
| T1 | Reference |  | Reference |  | Reference |  | Reference |  |
| T2 | 0.946(0.834-1.072) | 0.385 | 0.883(0.749-1.040) | 0.136 | 0.932(0.808-1.074) | 0.330 | 0.886(0.737-1.065) | 0.196 |
| T3 | 1.218(1.099-1.350 | **<0.001** | 0.955(0.804-1.134) | 0.600 | 1.265(1.126-1.420) | **<0.001** | 0.978(0.809-1.182) | 0.817 |
| T4 | 1.525(1.197-1.793) | **<0.001** | 1.049(0.837-1.316) | 0.677 | 1.629(1.362-1.949) | **<0.001** | 1.079(0.843-1.381) | 0.545 |
| N |  |  |  |  |  |  |  |  |
| N0 | Reference |  | Reference |  | Reference |  | Reference |  |
| N1 | 1.319(1.229-1.416) | **<0.001** | 1.127(0.985-1.290) | 0.083 | 1.413(1.305-1.530) | **<0.001** | 1.190(1.026-1.380) | **0.021** |
| N2 | 1.597(1.381-1.847) | **<0.001** | 1.495(1.221-1.831) | **<0.001** | 1.724(1.474-2.017) | **<0.001** | 1.609(1.292-2.003) | **<0.001** |
| N3 | 2.510(2.002-3.146) | **<0.001** | 2.108(1.555-2.859) | **<0.001** | 2.915(2.305-3.687) | **<0.001** | 2.464(1.791-3.390) | **<0.001** |
| M |  |  |  |  |  |  |  |  |
| M0 | Reference |  | Reference |  | Reference |  | Reference |  |
| M1 | 1.594(1.430-1.778) | **<0.001** | 0.983(0.657-1.471) | 0.935 | 1.747(1.555-1.963) | **<0.001** | 1.062(0.695-1.622) | 0.782 |
| Stage |  |  |  |  |  |  |  |  |
| I | Reference |  | Reference |  | Reference |  | Reference |  |
| II | 1.169(1.014-1.348) | **0.032** | 1.176(0.930-1.487) | 0.176 | 1.148(0.975-1.351) | 0.098 | 1.117(0.856-1.459) | 0.415 |
| III | 1.590(1.385-1.825) | **<0.001** | 1.379(1.033-1.841) | **0.029** | 1.701(1.454-1.991) | **<0.001** | 1.356(0.981-1.876) | 0.066 |
| IV | 2.256(1.917-2.656) | **<0.001** | 1.723(1.085-2.735) | **0.021** | 2.567(2.139-3.079) | **<0.001** | 1.648(1.002-2.713) | **0.049** |
| Grade |  |  |  |  |  |  |  |  |
| Grade I | Reference |  | Reference |  | Reference |  | Reference |  |
| Grade II | 1.099(0.935-1.291) | 0.252 | 1.068(0.908-1.255) | 0.429 | 1.102(0.919-1.322) | 0.294 | 1.077(0.897-1.292) | 0.427 |
| Grade III | 1.387(1.182-1.627) | **<0.001** | 1.308(1.114-1.536) | **0.001** | 1.462(1.221-1.750) | **<0.001** | 1.388(1.159-1.663) | **<0.001** |
| Grade IV | 1.441(1.036-2.004) | **0.030** | 1.257(0.902-1.751) | 0.176 | 1.418(0.972-2.070) | 0.070 | 1.221(0.836-1.785) | 0.302 |
| Therapy |  |  | Not selected |  |  |  | Not selected |  |
| No Adjuvant Therapy | Reference |  |  |  | Reference |  |  |  |
| Adjuvant Therapy | 0.995(0.912-1.087) | 0.919 |  |  | 1.083(0.986-1.191) | 0.096 |  |  |

Supplemental table 2 Univariate and multivariate analyses of overall survival (OS) and cancer-specific survival (CSS) in esophageal cancer patients after NAT before PSM

|  | OS(N=2136) | | | | CSS(N=2136) | | | |
| --- | --- | --- | --- | --- | --- | --- | --- | --- |
|  | Univariate | | Multivariate | | Univariate | | Multivariate | |
| Characteristics | HR（95%CI） | P | HR（95%CI） | P | HR（95%CI） | P | HR（95%CI） | P |
| Sex |  |  |  |  |  |  |  |  |
| Female | Reference |  | Reference |  | Reference |  | Reference |  |
| Male | 1.300(1.071-1.578) | **0.008** | 1.184(0.967-1.449) | 0.102 | 1.303(1.055-1.609) | **0.014** | 1.121(0.900-1.396) | 0.307 |
| Age |  |  | Not selected |  |  |  | Not selected |  |
| <60 | Reference |  |  |  | Reference |  |  |  |
| ≥60 | 0.968(0.862-1.088) | 0.586 |  |  | 0.934(0.823-1.060) | 0.292 |  |  |
| Race |  |  |  |  |  |  | Not selected |  |
| Black | Reference |  | Reference |  | Reference |  |  |  |
| White | 0.686(0.515-0.913) | **0.010** | 0.686(0.510-0.922) | **0.012** | 0.832(0.594-1.167) | 0.287 |  |  |
| Other | 0.545(0.367-0.808) | **0.003** | 0.541(0.362-0.808) | **0.003** | 0.694(0.445-1.082) | 0.107 |  |  |
| Year of diagnosis |  |  |  |  |  |  |  |  |
| 2007-2014 | Reference |  | Reference |  | Reference |  | Reference |  |
| 2015-2021 | 0.821(0.729-0.925) | **0.001** | 0.807(0.705-0.924) | **0.002** | 0.789(0.694-0.896) | **<0.001** | 0.749(0.646-0.867) | **<0.001** |
| Histologic Type ICD-O-3 |  |  |  |  |  |  |  |  |
| Adenocarcinoma | Reference |  | Reference |  | Reference |  | Reference |  |
| Squamous cell carcinoma | 0.839(0.707-0.995) | **0.044** | 0.873(0.717-1.063) | 0.176 | 0.811(0.671-0.979) | **0.029** | 0.933(0.768-1.134) | 0.485 |
| Other | 1.203(0.870-1.663) | 0.264 | 1.031(0.741-1.435) | 0.855 | 1.247(0.881-1.766) | 0.213 | 1.066(0.749-1.518) | 0.721 |
| Primary Site-labeled |  |  |  |  |  |  | Not selected |  |
| Lower third of esophagus | Reference |  | Reference |  | Reference |  |  |  |
| Middle third of esophagus | 0.984(0.794-1.218) | 0.879 | 1.176(0.928-1.491) | 0.179 | 0.914(0.721-1.159) | 0.458 |  |  |
| Other | 1.373(1.128-1.670) | **0.002** | 1.403(1.148-1.715) | **0.001** | 1.190(0.950-1.491) | 0.130 |  |  |
| Combined Summary Stage |  |  |  |  |  |  |  |  |
| Distant | Reference |  | Reference |  | Reference |  | Reference |  |
| Localized | 0.475(0.378-0.598) | **<0.001** | 0.763(0.501-1.160) | 0.205 | 0.432(0.334-0.558) | **<0.001** | 0.785(0.496-1.241) | 0.300 |
| Regional | 0.725(0.628-0.837) | **<0.001** | 0.837(0.666-1.053) | 0.129 | 0.712(0.609-0.831) | **<0.001** | 0.848(0.661-1.086) | 0.191 |
| Sequence number |  |  |  |  |  |  |  |  |
| One primary only | Reference |  | Reference |  | Reference |  | Reference |  |
| ≥2 primaries | 0.842(0.735-0.965) | **0.013** | 0.897(0.780-1.032) | 0.127 | 0.727(0.623-0.848) | **<0.001** | 0.801(0.684-0.938) | **0.006** |
| T |  |  |  |  |  |  |  |  |
| T1 | Reference |  | Reference |  | Reference |  | Reference |  |
| T2 | 0.875(0.690-1.110) | 0.273 | 0.811(0.604-1.091) | 0.166 | 0.937(0.718-1.224) | 0.635 | 0.837(0.603-1.163) | 0.290 |
| T3 | 1.212(1.008-1.459) | **0.041** | 0.821(0.620-1.086) | 0.167 | 1.342(1.088-1.656) | **0.006** | 0.869(0.638-1.184) | 0.374 |
| T4 | 1.361(1.025-1.806) | **0.033** | 0.824(0.568-1.195) | 0.307 | 1.468(1.071-2.014) | **0.017** | 0.832(0.553-1.250) | 0.375 |
| N |  |  |  |  |  |  |  |  |
| N0 | Reference |  | Reference |  | Reference |  | Reference |  |
| N1 | 1.348(1.179-1.540) | **<0.001** | 1.058(0.850-1.318) | 0.613 | 1.393(1.201-1.614) | **<0.001** | 1.063(0.837-1.350) | 0.617 |
| N2 | 1.503(1.201-1.880) | **<0.001** | 1.289(0.941-1.766) | 0.114 | 1.628(1.283-2.066) | **<0.001** | 1.442(1.028-2.025) | **0.034** |
| N3 | 1.519(1.017-2.268) | **0.041** | 1.241(0.757-2.036) | 0.392 | 1,670(1.097-2.541) | **0.017** | 1.369(0.813-2.307) | 0.237 |
| M |  |  |  |  |  |  |  |  |
| M0 | Reference |  | Reference |  | Reference |  | Reference |  |
| M1 | 1.499(1.267-1.773) | **<0.001** | 0.876(0.428-1.790) | 0.716 | 1.558(1.300-1.866) | **<0.001** | 0.917(0.432-1.950) | 0.822 |
| Stage |  |  |  |  |  |  |  |  |
| I | Reference |  | Reference |  | Reference |  | Reference |  |
| II | 1.172(0.882-1.559) | 0.273 | 1.217(0.757-1.955) | 0.418 | 1.309(0.939-1.825) | 0.113 | 1.269(0.744-2.163) | 0.382 |
| III | 1.670(1.274-2.190) | **<0.001** | 1.664(0.957-2.892) | 0.071 | 1.940(1.413-2.663) | **<0.001** | 1.740(0.939-3.226) | 0.079 |
| IV | 2.164(1.602-2.923) | **<0.001** | 1.997(0.843-4.731) | 0.116 | 2.575(1.821-3.641) | **<0.001** | 2.098(0.827-5.322) | 0.119 |
| Grade |  |  |  |  |  |  |  |  |
| Grade I | Reference |  | Reference |  | Reference |  | Reference |  |
| Grade II | 1.011(0.764-1.337) | 0.940 | 0.984(0.742-1.304) | 0.908 | 0.992(0.728-1.351) | 0.959 | 0.994(0.728-1.357) | 0.969 |
| Grade III | 1.267(0.962-1.669) | 0.092 | 1.206(0.913-1.592) | 0.187 | 1.345(0.993-1.822) | 0.056 | 1.311(0.966-1.781) | 0.083 |
| Grade IV | 2.239(1.329-3.772) | **0.002** | 2.086(1.229-3.540) | **0.006** | 2.299(1.299-4.067) | **0.004** | 2.007(1.128-3.570) | **0.018** |
| Therapy |  |  | Not selected |  |  |  | Not selected |  |
| No Adjuvant Therapy | Reference |  |  |  | Reference |  |  |  |
| Adjuvant Therapy | 0.981(0.876-1.100) | 0.746 |  |  | 1.058(0.934-1.197) | 0.375 |  |  |

Supplemental table 3 Univariate and multivariate analyses of OS and CSS in esophageal cancer patients who received neoadjuvant therapy after PSM

| Therapy Group | Preoperative Treatment | Postoperative Treatment | n=6141 | 2007-2014  （n=3078） | 2015-2021  (N=3063) | P |
| --- | --- | --- | --- | --- | --- | --- |
| No Adjuvant Therapy (N=5025) | Systemic Therapy | / | 307 | 199（6.47%） | 108(3.53%) | <0.001 |
|  | Radiation Therapy | / | 39 | 24（0.78%） | 15(0.49%) | 0.153 |
|  | Systemic Therapy + Radiation Therapy | / | 4679 | 2404(78.10%) | 2275(74.28%) | <0.001 |
| Adjuvant Therapy (N=1116) | Systemic Therapy | Systemic Therapy | 114 | 55(1.79%) | 59(1.93%) | 0.686 |
|  | Systemic Therapy | Radiation Therapy | 92 | 63(2.05%) | 29(0.95%) | <0.001 |
|  | Systemic Therapy | Systemic Therapy+Radiation Therapy | 46 | 27(0.88%) | 19(0.62%) | 0.243 |
|  | Radiation Therapy | Systemic Therapy | 61 | 29(0.94%) | 32(1.04%) | 0.685 |
|  | Radiation Therapy | Radiation Therapy | 4 | 3(0.10%) | 1(0.03%) | 0.620 |
|  | Radiation Therapy | Systemic Therapy+Radiation Therapy | 10 | 4(0.13%) | 6(0.20%) | 0.746 |
|  | Systemic Therapy+Radiation Therapy | Systemic Therapy | 575 | 172(5.59%) | 403(13.16%) | <0.001 |
|  | Systemic Therapy+Radiation Therapy | Radiation Therapy | 79 | 35(1.14%) | 44(1.44%) | 0.298 |
|  | Systemic Therapy+Radiation Therapy | Systemic Therapy+Radiation Therapy | 135 | 63(2.05%) | 72(2.35%) | 0.417 |

Supplemental table 4 Overall and subgroup-specific details of all treatment modalities are presented in this table. Data are expressed as n (%). Differences in treatment modality distribution between the 2007–2014 and 2015–2021 cohorts were compared using the Chi-square test.

|  | Adjuvant Systemic  Therapy  (n=114) | Adjuvant Radiotherapy  (n=92) | Adjuvant Systemic Therapy + Radiotherapy  (n=46) | No Adjuvant Therapy  (N=307) |
| --- | --- | --- | --- | --- |
| Sex, No. (%) |  |  |  |  |
| Female | 13 (11.40%) | 18 (19.57%) | 8 (17.39%) | 49 (15.96%) |
| Male | 101 (88.60%) | 74 (80.43%) | 38 (82.61%) | 258 (84.04%) |
| Age, No. (%) |  |  |  |  |
| <60 | 50 (43.86%) | 34 (36.96%) | 16 (34.78%) | 98 (31.92%) |
| ≥60 | 64 (56.14%) | 58 (63.04%) | 30 (65.22%) | 209 (68.08%) |
| Race |  |  |  |  |
| Black | 5 (4.39%) | 8 (8.70%) | 0 (0.00%) | 17 (5.54%) |
| White | 98 (85.96%) | 76 (82.61%) | 43 (93.48%) | 279 (90.88%) |
| Other | 11 (9.65%) | 8 (8.70%) | 3 (6.52%) | 11 (3.58%) |
| Year of diagnosis |  |  |  |  |
| 2007-2014 | 55 (48.25%) | 63 (68.48%) | 27 (58.70%) | 199 (64.82%) |
| 2015-2021 | 59 (51.75%) | 29 (31.52%) | 19 (41.30%) | 108 (35.18%) |
| Histologic Type ICD-O-3 |  |  |  |  |
| Adenocarcinoma | 101 (88.60%) | 65 (70.65%) | 36 (78.26%) | 254 (82.74%) |
| Squamous cell carcinoma | 10 (8.77%) | 26 (28.26%) | 8 (17.39%) | 45 (14.66%) |
| Other | 3 (2.63%) | 1 (1.09%) | 2 (4.35%) | 8 (2.61%) |
| Primary Site-labeled |  |  |  |  |
| Lower third of esophagus | 102 (89.47%) | 71 (77.17%) | 34 (73.91%) | 234 (76.22%) |
| Middle third of esophagus | 4 (3.51%) | 12 (13.04%) | 7 (15.22%) | 18 (5.86%) |
| Other | 8 (7.02%) | 9 (9.78%) | 5 (10.87%) | 55 (17.92%) |
| Combined Summary Stage |  |  |  |  |
| Distant | 17 (14.91%) | 18 (19.57%) | 6 (13.04%) | 54 (17.59%) |
| Localized | 8 (7.02%) | 12 (13.04%) | 9 (19.57%) | 69 (22.48%) |
| Regional | 89 (78.07%) | 62 (67.39%) | 31 (67.39%) | 184 (59.93%) |
| Sequence number |  |  |  |  |
| One primary only | 85 (74.56%) | 67 (72.83%) | 33 (71.74%) | 224 (72.96%) |
| ≥2 primaries | 29 (25.44%) | 25 (27.17%) | 13 (28.26%) | 83 (27.04%) |
| T |  |  |  |  |
| T1 | 12 (10.53%) | 9 (9.78%) | 11 (23.91%) | 68 (22.15%) |
| T2 | 10 (8.77%) | 12 (13.04%) | 4 (8.70%) | 48 (15.64%) |
| T3 | 80 (70.18%) | 61 (66.30%) | 28 (60.87%) | 166 (54.07%) |
| T4 | 12 (10.53%) | 10 (10.87%) | 3 (6.52%) | 27 (8.80%) |
| N |  |  |  |  |
| N0 | 31 (27.19%) | 30 (32.61%) | 16 (34.78%) | 125 (40.72%) |
| N1 | 60 (52.63%) | 57 (61.96%) | 26 (56.52%) | 169 (55.05%) |
| N2 | 14 (12.28%) | 5 (5.43%) | 3 (6.52%) | 8 (2.61%) |
| N3 | 9 (7.89%) | 0 (0.00%) | 1 (2.17%) | 5 (1.63%) |
| M |  |  |  |  |
| M0 | 101 (88.60%) | 79 (85.87%) | 40 (86.96%) | 266 (86.65%) |
| M1 | 13 (11.40%) | 13 (14.13%) | 6 (13.04%) | 41 (13.35%) |
| Stage |  |  |  |  |
| I | 4 (3.51%) | 6 (6.52%) | 7 (15.22%) | 47 (15.31%) |
| II | 32 (28.07%) | 26 (28.26%) | 9 (19.57%) | 90 (29.32%) |
| III | 57 (50.00%) | 45 (48.91%) | 23 (50.00%) | 124 (40.39%) |
| IV | 21 (18.42%) | 15 (16.30%) | 7 (15.22%) | 46 (14.98%) |
| Grade |  |  |  |  |
| Grade I | 6 (5.26%) | 4 (4.35%) | 0 (0.00%) | 16 (5.21%) |
| Grade II | 39 (34.21%) | 40 (43.48%) | 21 (45.65%) | 142 (46.25%) |
| Grade III | 68 (59.65%) | 47 (51.09%) | 24 (52.17%) | 144 (46.91%) |
| Grade IV | 1 (0.88%) | 1 (1.09%) | 1 (2.17%) | 5 (1.63%) |

Supplemental Table 5 The baseline characteristics of the four adjuvant treatment subgroups corresponding to Figure 4.
